# Antibiotic therapy as a risk factor for chronic residual and phantom limb pain after combat-related amputation: a 212-patient cohort study

**DOI:** 10.3389/fpain.2026.1715446

**Published:** 2026-03-18

**Authors:** Kateryna Ksenchyna, Oleh Ksenchyn, Oleksandr Nazarchuk, Dmytro Dmytriiev

**Affiliations:** National Pirogov Memorial Medical University, Vinnytsya, Ukraine

**Keywords:** antibiotic, chronic pain, limb amputation, neurotoxity, phantom limb pain, residual limb pain

## Abstract

**Background:**

Antibiotic (AB) therapy is standard in managing combat-related infections, particularly after traumatic limb amputations. However, prolonged or combined antibiotic regimens may contribute to neuroinflammatory processes that predispose patients to chronic post-amputation pain (ChPAP), which combines the consepts of chronic residual limb pain (RLP) and phantom limb pain (PLP).

**Objective:**

To investigate associations between antibiotic use (duration, type, and combination) and the development of RLP and PLP in post-amputation military patients.

**Methods:**

This retrospective cohort study evaluated 212 military personnel treated between 2022 and 2024 for traumatic amputations. Antibiotic regimens, pain intensity, type, and chronicity were analyzed.

**Results:**

Chronic RLP/PLP developed in 94 patients (44.3%). Prolonged antibiotic use (>21 days) and combined regimens (≥2 antibiotics) were) were related with increased ChPAP risk in limbs Neuropathic pain was predominant in patients exposed to fluoroquinolones or metronidazole.

**Conclusion:**

Extended and multi-agent antibiotic therapy was associated with ChPAP after combat-related limb amputation. Personalized antimicrobial stewardship and early pain screening are recommended in this high-risk population.

## Introduction

Combat-related amputations pose a high risk for the development of persistent pain syndromes, particularly chronic post-amputation pain (ChPAP) which combines the concepts of residual limb pain (RLP) and phantom limb pain (PLP). RLP was defined as pain localized in the amputated limb, typically associated with the surgical site or stump tissues. PLP was defined as painful sensations perceived in the absent (amputated) part of the limb, distinct from stump discomfort, and persisting beyond the acute postoperative phase. While traumatic injury, nerve damage, and psychological stress are established contributors, the role of medical interventions—especially systemic antibiotic (AB) therapy—has gained increasing attention.

Limb amputation creates conditions for altered perception and uncomfortable sensations in the lost body part, known as phantom limb pain (PLP). Residual limb pain (RLP), discomfort in the remaining body part, is also common. Amputation increases the risk of musculoskeletal disorders by at least twofold and pain by at least threefold compared to mild combat injuries (1).

Epidemiological data indicate that about 95% of amputees report pain of varying intensity, with approximately 80% experiencing PLP and 67% reporting RLP (2). Besides, it is mention, that pain intensity tends to increase within a year post-trauma, becoming chronic in about 10% of cases. Managing chronic PLP and/or RLP is complex, involving not only the physical absence of a body part but also neuroplasticity in the central nervous system and changes in the somatosensory system. Deafferentation (loss of sensory input due to amputation or injury) leads to neural pathway reorganization, contributing to pain. PLP can present with varied clinical manifestations and symptom severity. However, some studies, using functional MRI and diffusion tensor imaging, suggest no statistical link between cortical reorganization and PLP development or intensity, indicating other factors influence PLP and RLP (3). Factors complicating PLP include stump pain, diabetic amputation, lower limb amputation, and depression. Traumatic or surgical amputations have a higher PLP incidence than congenital ones (4–6). Individuals with diabetes and peripheral vascular diseases face a higher risk of repeated amputations, further increasing PLP risk (7, 8).

Knowledge of optimal stump conditions and specific amputation techniques is crucial for successful rehabilitation. Biomechanical considerations of the stump are as important as the amputation's cause in selecting surgical techniques (9, 10).

Recent studies report positive outcomes in treating amputees with PLP using advanced surgical methods, but there's a need to evaluate and develop alternative approaches tailored to individual characteristics, particularly pain causes (11).

During ongoing global armed conflicts, treatment algorithms for traumatic injuries, including amputations, have improved. Emergency care and life-saving interventions are now available closer to front lines, with early antibiotic use and rapid evacuation to higher-level medical facilities improving survival rates. However, prolonged use of moderate to high antibiotic doses may lead to adverse effects (12).

Beta-lactams and fluoroquinolones are frequently cited for adverse effects. Rising microbial resistance forces clinicians to use higher doses, with neurotoxicity increasingly noted over the past decade due to prolonged antibiotic therapy (13, 14).

Antibiotics can affect the nervous system in two ways: through direct neurotoxic effects and indirect modulation of immune and microbiome-mediated pathways. Several classes of antimicrobial drugs, including fluoroquinolones, nitroimidazoles (e.g., metronidazole), and aminoglycosides, are increasingly recognized as “neuroactive antibiotics,” defined as agents that can cause neurological or neuropsychiatric side effects, including peripheral neuropathy, encephalopathy, and alterations in neurotransmitter signaling. Fluoroquinolones, widely used for their broad-spectrum action, are linked to higher risks of central nervous system and gastrointestinal issues compared to other antimicrobials (15, 16). Metronidazole is often associated with neuropathy and mitochondrial dysfunction, and aminoglycosides are implicated as a factor in oxidative stress and neuromuscular toxicity. The literature suggests that *β*-lactam antibiotics, especially at high and/or prolonged doses, may exhibit neurotoxic properties through interference with inhibitory neurotransmission. Antibiotics are often administered for prolonged periods due to contaminated wounds and osteomyelitis. Most studies on chronic pain after limb amputation focus on trauma-related mechanisms such as nerve injury, neuroplastic changes, or psychological factors. However, the contribution of pharmacotherapy—particularly antibiotic therapy—remains underexplored. The potential neurotoxic, neuroinflammatory, and microbiome-disrupting effects of prolonged or combined antibiotic use may represent an overlooked factor in pain chronification (17–19).

This study explores whether certain AB regimens (particularly combination therapy or extended courses) contribute to the risk of chronic pain in the residual limb or phantom sensations after limb amputation in military trauma patients.

## Materials and methods

We conducted a retrospective cohort study at three military referral hospitals in Ukraine between March 2022 and December 2024.

We performed the study in accordance with the World Medical Association Declaration of Helsinki on the ethical principles for medical research involving human subjects, the Council of Europe Convention on the Human Rights and Biomedicine, relevant laws, orders of the Ministry of Health of Ukraine. The local Committee on Bioethics of National Pirogov Memorial Medical University, Vinnytsya, Ukraine approved all steps of the research and the publication of the results was satisfied (Protocol No. 2; 10.02.2025).

### Participants

**Figure 1:**
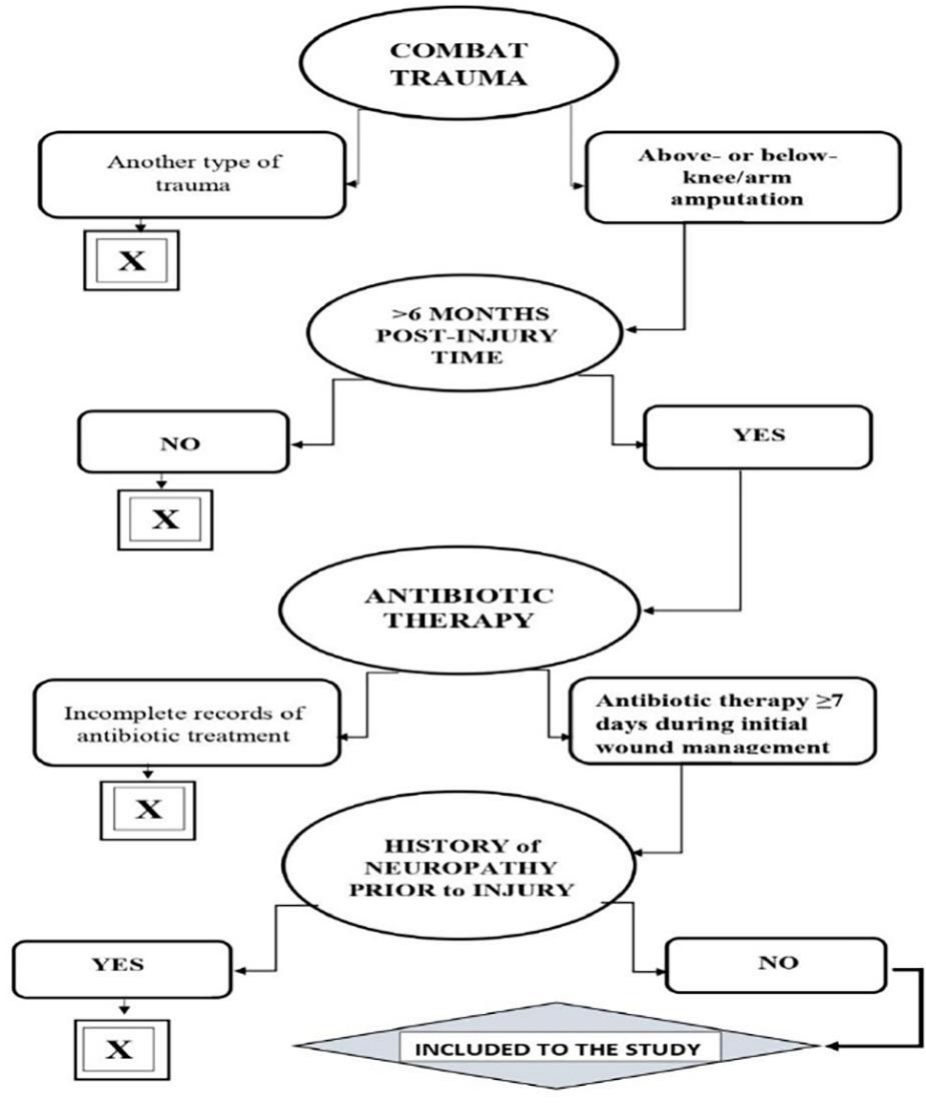
Inclusion criteria to the study.

We included 212 consecutive adult patients (aged 19–56) with traumatic limb amputation due to combat injuries. All participants were men. After assessing the inclusion and exclusion criteria, it was mandatory for each participant to be familiarized with the research methodology and for the participant to sign an informed consent to participate in it (Figure 1).

### Data collection

For each patient, the following data were recorded: age, sex, level of amputation (e.g., below-knee, above-knee, transhumeral, etc.), and the severity of the initial injury. The level of amputation was categorized as major (above-knee, through-knee, above-elbow, shoulder disarticulation) or minor [below-knee, Syme (ankle disarticulation), transradial, hand or digit amputations]. To minimize the factor related to the severity of infection, the exclusion group included patients with osteomyelitis and the need for surgical revision of the wound. In addition, we used the colony forming unit (CFU) value of the isolated bacterial isolates > 10^6^ as a criterion for assessing the severity of infection. We used the severity of infection as a criterion in the cluster analysis (Table 1).

**Table 1:** Characteristic of participants.

Characteristic	Value
Total patients	212
Mean age (±SD)	34.9 ± 7.3 years
Male	91%
Lower limb amputations	73%
Primary wound infection	58%
Antibiotic duration >21 d	38% (*n* = 81)
Combination AB therapy	64% (*n* = 136)

Additionally, information regarding antibiotics received by patients was documented: type of antibiotic (drug name), dosage, route of administration (oral, intravenous), and duration of use (in days). All study participants received one of the following antibiotics: beta-lactams (cephalosporins), aminoglycosides, fluoroquinolones, nitroimidazoles (metronidazole), and oxazolidinones (lizenolid). Patients were stratified by the duration of antibacterial therapy into three groups: ≤14 days, 15–21 days, and >21 days. The number of antibiotics was also considered: monotherapy (one drug) or combination therapy (two or more antibiotics simultaneously).

### Pain assessment

Pain syndrome was evaluated based on several parameters and classified as nociceptive, neuropathic, or mixed.

Pain intensity was initially assessed using the Numerical Rating Scale (NRS) at three follow-up visits: 3, 6, and 12 months post-amputation. The NRS rates pain intensity from 0 to 10, where 0 indicates no pain and 10 represents maximum pain.

The presence of neuropathic pain was assessed using the Douleur Neuropathique questionnaire (DN-4), with a total score of ≥4 indicating a positive result. The questionnaire consists of 10 items: 7 items related to the patient's subjective sensations (symptoms) and 3 items based on neurological examination findings (Bouhassira et al., 2005, https://doi.org/10.1016/j.pain.2004.12.010).

Pain duration was classified as acute (<3 months), subacute (3–6 months), or chronic (>6 months). Additionally, the presence of persistent pain was evaluated 12 months post-surgery.

### Statistical аnalysis

Descriptive statistics were applied to summarize demographic data. Categorical variables were analyzed using the chi-square test, while continuous variables were compared using one-way analysis of variance (ANOVA). To explore interrelationships among antibiotic-use characteristics and clinical covariates, we performed a principal component analysis (PCA) followed by cluster analysis.

Variables included in the PCA comprised the number of antibiotics, therapy duration (days), and antibiotic classes administered. Additional clinical covariates, such as age, and amputation level, infection severity were also considered.

Before PCA, we standardized all continuous variables (z-score normalization). The number of retained components was determined using the Kaiser criterion (eigenvalues > 1) and a scree plot. Components explaining the largest proportion of the total variance were then selected. Next, we used the Ward minimum variance method with Euclidean distance to identify subgroups of patients with similar antibiotic regimens. The optimal number of clusters was selected using the silhouette coefficient and the discontinuity statistic.The rationale for using PCA before clustering was to reduce multicollinearity and increase the stability of the clustering results by summing correlated variables related to antibiotic use into independent principal components. Following clustering, groups were relabeled according to their antibiotic exposure intensity to improve clinical interpretability. Using hierarchical clustering using Ward's minimum variance, we identified two distinct clusters of patients based on antibiotic exposure profiles. The numerical indexing used by the statistical software (e.g., Cluster 0 and Cluster 1) does not imply ranking and has been standardized. To more clearly interpret the clinical effect, we renamed the clusters to Cluster 1 (high cumulative antibiotic exposure with a predominance of neuroactive agents) and Cluster 2 (lower exposure profile dominated by standard prophylactic regimens). The cluster originally indexed as “Cluster 0” in the statistical software corresponds to the lower-exposure group (Cluster 2) in this manuscript.

Finally, multivariate logistic regression was used to identify independent predictors of chronic pain, including antibiotic-related clusters and relevant clinical variables. The dependent variable was the presence of chronic pain (persistent pain >6 months post-amputation). Independent variables included antibiotic duration category (≤14, 15–21, >21 days), number of antibiotics (mono- vs. combination therapy), amputation type (major vs. minor), presence of infection, age, injury severity score, and level of amputation (upper vs. lower limb).

Covariate inclusion followed a two-step process: variables with *p* < 0.10 in univariate analyses or considered clinically relevant based on prior literature were entered into the multivariate model using a backward stepwise elimination method. A *p*-value < 0.05 was considered statistically significant.

## Results

In total, chronic pain lasting more than three months was detected in 94 of 212 examined individuals, which is 44.3% of the sample. A more detailed analysis of the distribution of chronic pain types showed the following. In 32 cases (out of 94), only residual limb pain (RLP) was observed. In 28 cases, only phantom limb pain (PLP). In 34 participants, both types of pain were present—both RLP and PLP. Thus, among patients with chronic pain, almost a third had a combined form of pain syndrome (36.2% out of 94), which may indicate more complex mechanisms of pathological nociception after amputation. Given the significant prevalence and variability of manifestations of chronic pain, these results emphasize the need for a differentiated approach to the diagnosis and treatment of patients with amputations.

We analyzed the relationship between the prevalence of chronic pain and the duration of antibiotic use and the number of antibiotics in the treatment regimen. According to the results of statistical calculations, it was found that patients who took antibiotics for more than 21 days had significantly more chronic pain (59.2%), compared with those who took them ≤14 days (27.1%), *p* < 0.001. Based on the assessment of the characteristics of chronic pain depending on the number of antibiotics the patient received, we obtained the following results: pain occurred more often in patients who received combination therapy (51.4%), compared with those who received monotherapy (33.7%), *p* = 0.004 (Table 2).

**Table 2:** The relationship between antibiotic therapy and the prevalence of chronic pain.

Group	Chronic pain prevalence	*p*-value
AB duration ≤14 days	27.1%	—
AB duration >21 days	59.2%	<0.001
Monotherapy	33.7%	—
Combination therapy	51.4%	0.004

Given these results, the next step in our calculation was to use logistic regression to test whether criteria such as duration of antibiotic therapy, combination regimen, or type of antibiotic were independent predictors of chronic pain. In particular, we found that duration of antibiotic therapy of more than 21 days was associated with a higher risk of chronic pain compared with regimens of up to 21 days by almost threefold (OR = 2.89; 95% CI: 1.64–5.10; *p* < 0.001). In addition, combination antibiotic therapy was also an independent risk factor for chronic pain (OR = 1.83; 95% CI: 1.07–3.13; *p* = 0.027). Among all types of antibacterial drugs in our study, the use of fluoroquinolones was associated with an increased risk of chronic pain by more than twofold (OR = 2.42; 95% CI: 1.13–5.21; *p* = 0.021). At the same time, assessing the impact of the severity of the initial infection, there was a trend towards an increased risk of chronic pain in patients with severe infection (*p* = 0.115) (Table 3).

**Table 3:** Independent predictors of the development of chronic pain.

Predictor	OR (95% CI)	*p*-value
AB duration >21 days	2.89 (1.64–5.10)	<0.001
Combination AB therapy	1.83 (1.07–3.13)	0.027
Fluoroquinolone use	2.42 (1.13–5.21)	0.021
Severe initial infection	1.52 (0.89–2.60)	0.115

### Neuropathic pain characteristics

Among 94 patients with chronic pain, neuropathic symptoms (positive result on the DN4 scale) were detected in 61 individuals (65%). Analysis of the association with individual classes of antibacterial drugs showed the following: among patients receiving fluoroquinolones (*n* = 28), neuropathic pain was recorded in 21 cases (75%); with metronidazole (*n* = 19)—in 15 patients (79%). In the group receiving cephalosporins (*n* = 74), neuropathic pain was detected in 36% of cases. In addition, the use of more than two classes of antibiotics was associated with higher pain intensity (*p* = 0.03).

**Figure 2:**
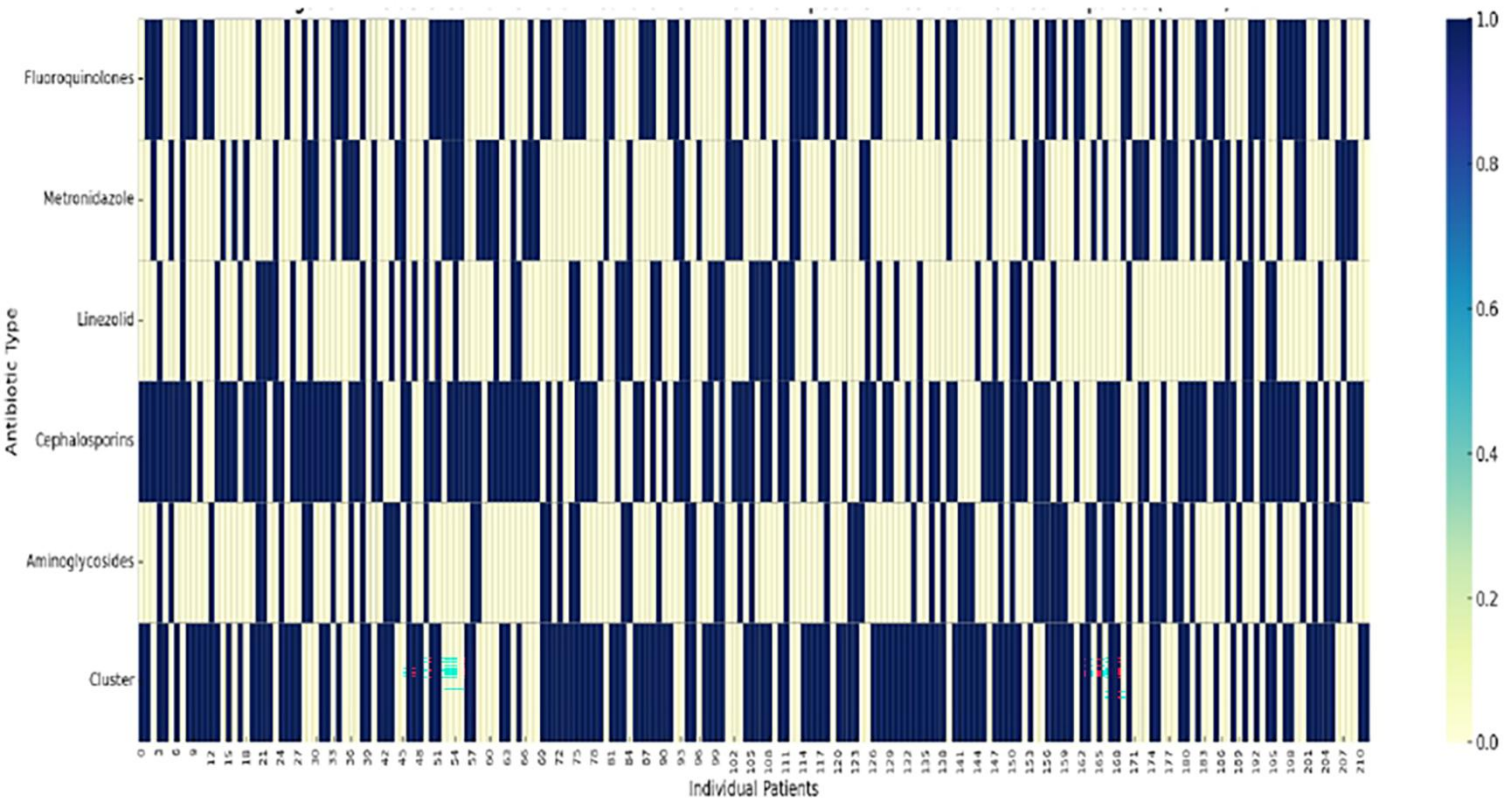
Clustered patterns of neurotoxic antibiotic exposure in combat-related amputees (*n* = 212). This heatmap illustrates binary exposure (yes/no) to key neurotoxic antibiotics (rows) across 212 patients with combat-related amputations (columns). The bottom row shows unsupervised clustering results, highlighting distinct antibiotic exposure profiles. The cluster originally indexed as “Cluster 0” in the statistical software corresponds to the lower-exposure group (Cluster 2) in this manuscript.

We revealed two distinct clusters of patients based on their neurotoxic antibiotic exposure profiles. Cluster 1 (high antibiotic exposure cluster) is defined by frequent use of fluoroquinolones, metronidazole, and aminoglycosides, indicating a high-risk group for neuroinflammation and chronic pain. Wound care appears to contribute to these profiles but does not independently drive clustering. Cluster 2 represented a lower exposure cluster, predominantly associated with standard prophylactic regimens and shorter courses of antibiotics. Conversely, linezolid use, although less frequent, co-segregates with the high-exposure cluster, suggesting a potential cumulative neurotoxic burden (Figure 2).

**Figure 3:**
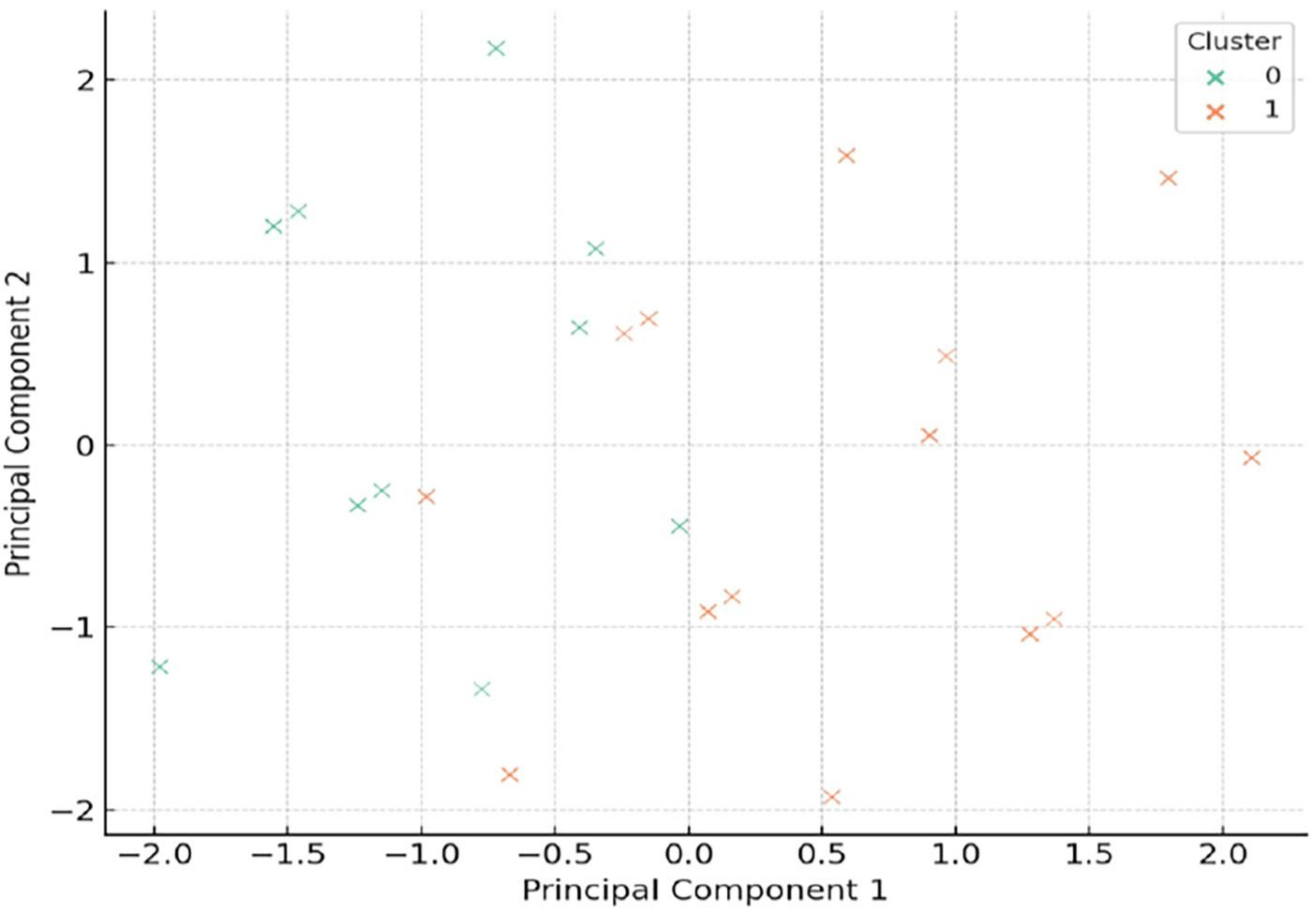
Clustered patterns of neurotoxic antibiotic exposure in combat-related amputees (*n* = 212). This graph displays the binary exposure (present/absent) to five classes of neurotoxic antibiotics across 212 combat-injured patients who underwent limb amputation. Patients (columns) were grouped using unsupervised clustering based on antibiotic exposure patterns. Cluster 1 (high antibiotic exposure cluster) is characterized by the frequent co-administration of fluoroquinolones, metronidazole, and aminoglycosides—agents known for neurotoxicity and glial activation. This cluster likely represents a high-risk subgroup for developing chronic residual limb or phantom limb pain.

Using PCA-based dimensionality reduction, we visualized high-dimensional antibiotic exposure data in a simplified two-dimensional space, revealing latent patterns in prescribing practices among combat-injured amputees. The analysis identifies a distinct patient subset (Cluster 1) characterized by frequent, combined exposure to neurotoxic antibiotics, including fluoroquinolones, metronidazole, and aminoglycosides (Figure 3).

**Figure 4:**
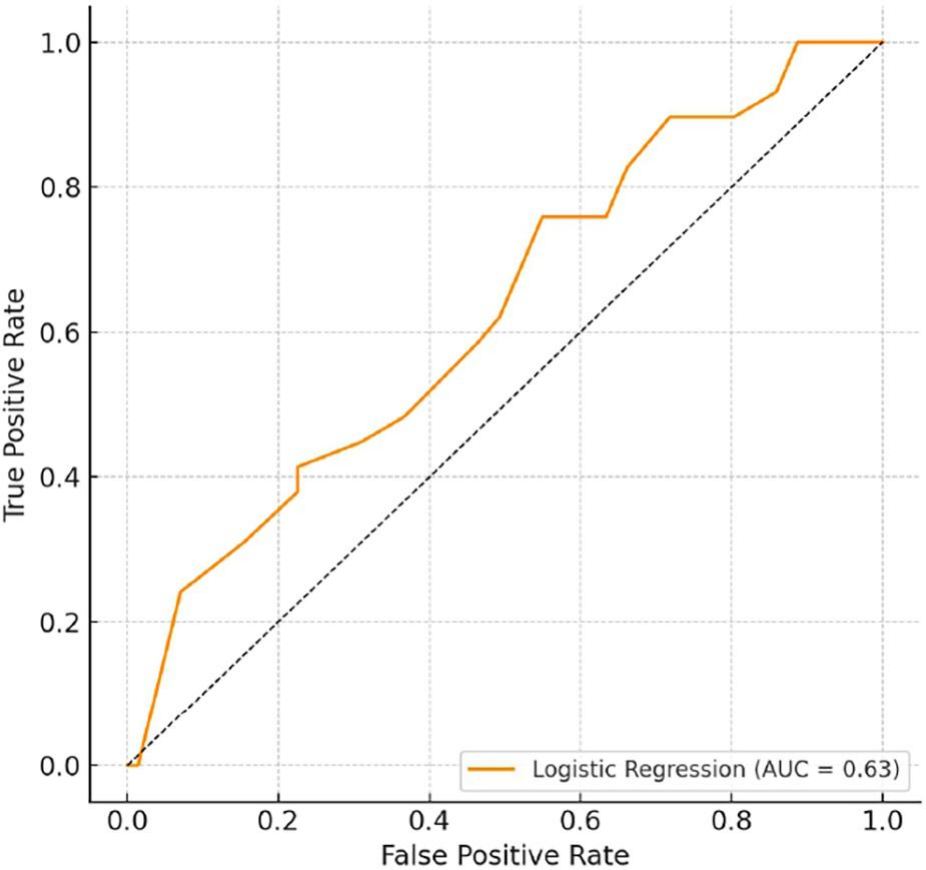
ROC curve for chronic pain prediction. Receiver Operating Characteristic (ROC) curve for a logistic regression model trained on antibiotic exposure features to predict chronic residual limb or phantom limb pain. The area under the curve (AUC = 0.63) indicates moderate discriminatory ability of antibiotic exposure profiles in identifying patients at risk for chronic pain.

An AUC of 0.63 indicates moderate predictive value, suggesting that antibiotic exposure profiles provide some insight into identifying patients at risk of pain chronification, though they are not robust enough for use as a standalone diagnostic tool (Figure 4).

## Discussion

In recent years, increasing attention has been paid to the theory of the functioning of the microbiome–gut–brain axis and the role of glial cells in the modulation of the neuroimmune response under the influence of the microbiota. Evidence suggests that the microbiome can indirectly influence the activity of microglia and astrocytes through metabolic products (e.g., short-chain fatty acids) or by stimulating intestinal immune cells, which, in turn, changes the systemic cytokine profile. Activation of glial cells can contribute to both neuroprotection and the development of neuroinflammation, depending on the context of the signals. These mechanisms emphasize the importance of a holistic approach to studying the gut–brain axis, which takes into account not only neuronal, but also immune and glial components that interact in a complex network of interorgan communication. In this context, the study of the consequences of the use of antibiotics, which can change the composition and functional activity of the microbiota, which, in turn, can affect glial activation and modulation of pain sensitivity, is of particular importance (20, 21).

This study demonstrates a significant association between prolonged and combined antibiotic therapy and the development of chronic pain (RLP/PLP) in combat-related amputations. Nearly half of all patients developed chronic pain, and neuropathic components predominated among them. Our results are consistent with previous preclinical data suggesting that fluoroquinolones, metronidazole, and other neuroactive antibiotics may enhance microglial activation, mitochondrial dysfunction, or disrupt neuroimmune signaling. In addition, prolonged antibiotic exposure likely contributes to gut dysbiosis, altering gut-brain axis interactions, and systemic inflammation—both of which are associated with chronic pain.

For example, in their work, researchers Hurkacz et al. also conducted a detailed analysis of the possibility of neurotoxicity as an undesirable effect of antibiotics. In their article, they consider metronidazole and fluoroquinolones among different classes of antibiotics as those most often associated with neurotoxic effects. This coincided with the result obtained in our study (22).

Other studies, such as a systematic review by Maan et al., which included 92 articles, found that high-intensity cefepime caused altered mental status, myoclonus, and nonconvulsive seizures. Furthermore, these symptoms resolved after dose reduction or discontinuation of the antibiotic (23).

We did not study cefepime in the study cohort, however, it is often mentioned in the literature as a representative *β*-lactam associated with neurotoxicity. For example, a 10-year systematic review of the neurotoxicity of cefepime, conducted in 2023 by Ajibola et al, concluded that cefepime is an effective antibiotic against Gram-negative bacteria such as Pseudomonas aeruginosa, but caution is needed in its use. It is worth noting that the mechanism of cefepime-induced neurotoxicity remains unknown. Hypotheses put forward for this phenomenon consider the antibiotic's ability to cross the blood-brain barrier and cause an imbalance in neurotransmitter functions (24).

Importantly, combination therapy and regimens longer than 3 weeks appear to pose the highest risk, even after controlling for injury severity.

Notably, the cluster analysis includes patients with multiple concurrent exposures to agents known to impair mitochondrial function, activate glial cells, and induce peripheral neuropathy. The widespread concomitant use of fluoroquinolones and metronidazole in this group is consistent with preclinical data showing increased microglial activation and increased oxidative stress in peripheral nerves. In addition, the frequent use of cephalosporins reflects their role as standard prophylaxis in combat settings. The unsupervised clustering approach confirms the presence of at least one subgroup of patients with a neurotoxic signature of exposure. This finding may have prognostic implications: patients in Cluster 1 may require more intensive monitoring for early signs of residual limb pain (RLP), phantom limb pain (PLP), or sensory disturbances., metronidazole, and aminoglycosides are known to impair mitochondrial function, induce oxidative stress, and activate neuroimmune pathways such as glial cell reactivity.

In addition, Barberán J et al. in their work indicate that, the FDA received 210,705 adverse event reports for marketed fluoroquinolones between 1997 and 2015. The most commonly reported toxicities were neurologic (30% and 26%) (15).

Importantly, the clusters were generated independently of clinical pain outcomes using only binary exposure data, but the emergence of a cluster with high exposure provides strong evidence for an underlying phenotype that may be biologically and clinically relevant. Patients in Cluster 1 may represent a neurobiologically vulnerable population predisposed to persistent pain due to the cumulative neurotoxic and pro-inflammatory effects of their antibiotic therapy.

This unsupervised method also demonstrates potential utility for early risk stratification: identifying patients at increased risk of developing chronic limb pain (CLP) or phantom limb pain (PLP) based solely on treatment history. In future practice, such clustering obtained using PCA may aid clinical decision-making by informing the intensity of follow-up, early referral to pain specialists, or modification of antimicrobial protocols to mitigate long-term neurotoxic consequences. The AUC value of 0.63 indicates moderate predictive value, indicating that antibiotic exposure profiles carry some informative signal for identifying patients at risk of chronic pain, although not strong enough to be used as a stand-alone diagnostic tool. This modest efficacy may reflect the multifactorial nature of chronic post-amputation pain, which, in addition to pharmacological exposure, is influenced by injury severity, nerve injury, psychological factors, and prosthetic use.

Nevertheless, the result supports the biological plausibility that cumulative or specific neurotoxic antibiotic regimens contribute to vulnerability to pain, and highlights the potential role of antibiotic history as a component in complex risk models. Future studies integrating additional clinical and biological data (e.g., pain phenotypes, inflammatory markers, microbiome profiles) may significantly improve the performance of the model.

Thus, the observed separation between clusters in the PCA space is not simply a statistical artifact, but likely reflects clinically actionable patterns of pharmacological action, highlighting the need to integrate antimicrobial stewardship with long-term pain monitoring in military medicine.

Limitations include the retrospective design, reliance on record accuracy, and potential unmeasured factors influencing outcome (e.g., psychological trauma, prosthetic use, access to rehabilitation).

## Conclusions

Prolonged and combined antibiotic therapy may be an under-recognized factor contributing to chronic residual and phantom limb pain in patients with combat-related amputations. Clinicians should consider minimizing unnecessary AB duration and monitor for early signs of neuropathic pain, especially in patients receiving fluoroquinolones or metronidazole. Future prospective studies should further delineate associative mechanisms and protective strategies.

## Data Availability

The raw data supporting the conclusions of this article will be made available by the authors, without undue reservation.
